# Development and comparison of machine learning models for predicting moderate-to-severe tinnitus in patients with hearing loss

**DOI:** 10.3389/fneur.2025.1741302

**Published:** 2026-01-12

**Authors:** Chenguang Zhang, Tao Ran, Yicong Wang, Di Xiao, Yuwen Wang, Ying Zhang, Ying Zhang, Bin Guo

**Affiliations:** 1 Qinghai University, Xining, China; 2 Department of Otolaryngology, Qinghai University Affiliated Hospital, Xining, China; 3 Department of Gastrointestinal Surgery, The Third Affiliated Hospital of Sun Yat-Sen University, Guangzhou, China; 4 Dalian Medical University, Dalian, China; 5 Zhejiang University School of Medicine, Hangzhou, China

**Keywords:** hearing loss, machine learning, random forest, sleep disorder, tinnitus

## Abstract

**Objective:**

Analyze the psychological and clinical factors of clinically significant tinnitus (THI score ≥38) in patients with hearing loss, construct predictive models based on four machine learning (ML) algorithms, and compare the predictive performance of different models.

**Methods:**

Patients with hearing loss who visited the Department of Otolaryngology at Qinghai University between August 2024 and May 2025 were enrolled in this study. Clinical data were retrieved from the hospital’s electronic medical record system. The study outcome was the occurrence of clinically significant tinnitus. Predictive variables were screened using univariate analysis, the least absolute shrinkage and selection operator (LASSO) regression, and the Boruta algorithm. Four ML algorithms—logistic regression (LR), random forest (RF), extreme gradient boosting (XGBoost), and support vector machine (SVM)—were applied to construct and validate predictive models. The area under the receiver operating characteristic curve (AUC) of each model in the validation set was compared using the DeLong test. Additionally, model performance metrics in the validation set were compared to identify the optimal model. Finally, the Shapley additive explanations (SHAP) algorithm was employed to interpret the best-performing model.

**Results:**

Nine key variables—age, hypertension, sleep disorder, anxiety, hearing loss severity, depression, noise exposure history, hearing side, and ototoxic drug use—were retained after LASSO and Boruta feature selection. Among the four ML models, the RF algorithm achieved the best predictive performance, with an AUC of 0.973 in the training set and 0.977 in the validation set, followed by XGBoost (AUC = 0.962 and 0.961, respectively). DeLong tests confirmed that RF significantly outperformed LR and SVM models (*p* < 0.001), while its difference from XGBoost was not significant. In the validation set, the RF model yielded the highest accuracy (0.923), sensitivity (0.929), specificity (0.914), precision (0.945), and F1-score (0.937). SHAP analysis indicated that hearing loss severity, age, and sleep disorder were the most influential predictors, suggesting that both auditory and non-auditory factors contribute substantially to the risk of clinically significant tinnitus.

**Conclusion:**

The RF model showed the best performance in predicting clinically significant tinnitus, with hearing loss severity, age, and sleep disorder identified as major predictors. Integrating auditory and psychological factors can improve early risk identification in patients with hearing loss.

## Introduction

Tinnitus, defined as the perception of sound without an external source, is a common auditory condition with significant global prevalence (1–4). Recent epidemiological estimates indicate that roughly 10–15% of adults experience tinnitus (3, 5). Prevalence increases with age—for example, it affects about 9–14% of middle-aged adults and rises to ~24% or more in those over 65 (5). While many cases are mild, tinnitus can be severe in a subset of patients: around 1–2% of the population suffers disabling tinnitus that substantially impairs quality of life (6). This translates to an estimated >740 million individuals affected worldwide, with over 120 million finding tinnitus to be a “major” problem (7). Such figures underscore the substantial public health burden of tinnitus.

Importantly, tinnitus is strongly associated with hearing loss. It often accompanies sensorineural hearing impairments (e.g., age-related or noise-induced hearing loss), and studies in older adults show an ~80% overlap between chronic tinnitus and measurable hearing deficits (8–10). Indeed, hearing damage (due to presbycusis, noise exposure, ototoxic drugs, etc.) is considered a primary trigger for tinnitus, as peripheral auditory deafferentation can induce aberrant neural activity perceived as “phantom” sound. Correspondingly, large-scale analyses confirm that hearing health is the single strongest predictor of tinnitus onset (11). At the same time, not everyone with hearing loss develops tinnitus, and some tinnitus patients have normal audiograms. This discrepancy suggests that factors beyond the auditory system modulate tinnitus susceptibility and severity (11, 12). In fact, recent genetic evidence indicates that while tinnitus and hearing loss share many risk variants, tinnitus has a distinct pathophysiology—including genetic influences and brain activity patterns—that cannot be explained by hearing loss alone. Tinnitus is now regarded as a cross-disciplinary disorder involving auditory, cognitive, and emotional components (7).

A robust body of research links tinnitus with psychological comorbidities. Many patients with chronic tinnitus experience elevated levels of anxiety, depressed mood, and sleep disturbances (3, 13). For instance, in a large national survey, 25.6% of tinnitus sufferers reported clinically significant depression in the past year, compared to only 9.1% in those without tinnitus (14). A meta-analysis likewise found a median ~33% prevalence of major depression among tinnitus patients (15). Anxiety disorders and symptoms are also frequently reported alongside tinnitus (13). Epidemiological studies and population-based cohorts have shown that even after adjusting for hearing status, individuals with tinnitus (including “non-bothersome” cases) are more likely to exhibit anxiety and depressive symptoms than those without tinnitus (16). Sleep problems are another well-documented companion of tinnitus: about 40–60% of tinnitus patients report poor sleep quality or insomnia, and sleep difficulties correlate with greater tinnitus distress (3). In fact, tinnitus is “not only correlated with hearing loss, but also with a spectrum of psychiatric disorders” including depression, anxiety, and even suicidal ideation in severe cases (7). These comorbid conditions can form a vicious cycle—persistent tinnitus provokes emotional distress and sleep disruption, which in turn can exacerbate the perception of tinnitus and its impact on well-being (11). In this study, we applied and compared multiple machine learning (ML) algorithms to develop predictive models for clinically significant tinnitus in patients with hearing loss, aiming to support early identification and personalized intervention.

## Materials and methods

### Population selection

Three hundred one patients with hearing loss who visited the Department of Otolaryngology, Qinghai University Affiliated Hospital, between August 2024 and May 2025 were retrospectively enrolled in this study. All participants were identified through the hospital’s electronic medical record system. Inclusion criteria were as follows: (1) age≥18 years; (2) completion of audiological assessments, detailed medical history, and psychological questionnaires; (3) completion of the Tinnitus Handicap Inventory (THI) for the evaluation of tinnitus severity; and (4) availability of all key clinical variables required for the analysis. Exclusion criteria included: (1) the presence of active acute ear diseases (e.g., otitis externa, otitis media, or sudden sensorineural hearing loss); (2) a history of cranial trauma or otologic surgery; (3) diagnosed neurodegenerative or severe psychiatric disorders; and (4) incomplete or missing clinical data.

### Data collection

Clinical data of all eligible patients were extracted from the hospital’s electronic medical record system by two independent researchers using a standardized data collection form. Demographic information included age, sex, and BMI. Clinical characteristics comprised hypertension, diabetes, hyperlipidemia, noise exposure history, family history of hearing loss, and the presence of sleep disorders, anxiety, or depression. Audiological data included the hearing loss severity, duration of hearing loss, and ototoxic drug use. Lifestyle factors such as smoking and alcohol consumption were also recorded. All psychological parameters were assessed using validated questionnaires administered at the time of evaluation. Tinnitus severity was quantified using the Tinnitus Handicap Inventory (THI) (17, 18), and patients with clinically significant tinnitus were identified based on THI scores. To ensure data integrity and consistency, all entries were cross-checked, and cases with missing or ambiguous information were excluded from the analysis.

### Definition of related variables

The primary outcome was tinnitus severity, assessed using the THI. According to standard grading criteria, THI scores were classified as follows: slight (0–16), mild (18–36), moderate (38–56), severe (58–76), and catastrophic (78–100). For analytical purposes, tinnitus severity was dichotomized as clinically significant tinnitus (THI score ≥38), which included moderate, severe, and catastrophic categories, versus no or mild tinnitus (THI score <38), representing the absence or minimal impact of tinnitus. The hearing loss severity was categorized based on pure-tone audiometry as mild (21–40 dB), moderate (41–60 dB), or severe (>60 dB) (19). Laryngopharyngeal reflux (LPR) was defined by a Reflux Symptom Index (RSI) ≥ 13 and a Reflux Finding Score (RFS) ≥ 7 (20, 21). Psychological variables included anxiety and depression, assessed using the Zung Self-Rating Anxiety Scale (SAS) and the Patient Health Questionnaire-9 (PHQ-9), respectively. Anxiety was defined as SAS ≥ 50, and depression as PHQ-9 ≥ 10 (22, 23). Sleep quality was evaluated using the Pittsburgh Sleep Quality Index (PSQI), with a total score >5 indicating poor sleep quality (24). Occupational noise exposure was defined as exposure to noise levels ≥85 dB for at least 8 h per day for a minimum duration of 1 year.

### Statistical methods

All statistical analyses were performed using R software (version 4.3.2). A total of 301 patients with hearing loss were included in the analysis. The dataset was randomly divided into a training set (80%) and a validation set (20%) to develop and evaluate predictive models. Continuous variables were expressed as mean ± standard deviation (SD) or median with interquartile range (IQR), depending on data distribution, and categorical variables were summarized as frequencies and percentages. Group comparisons were conducted using the t-test or Mann–Whitney U test for continuous variables and the chi-square or Fisher’s exact test for categorical variables.

The least absolute shrinkage and selection operator (LASSO) regression to penalize overfitting and identify variables with nonzero coefficients. In addition, the Boruta algorithm, a random-forest-based feature selection method, was applied to further validate the robustness of selected predictors. The final feature set was obtained by taking the union of variables identified by these two approaches.

Four supervised ML algorithms—logistic regression (LR), random forest (RF), extreme gradient boosting (XGBoost), and support vector machine (SVM)—were used to construct predictive models for clinically significant tinnitus in patients with hearing loss. Model performance was evaluated in both the training and validation sets. The area under the receiver operating characteristic curve (AUC) was calculated to assess discriminative ability, and DeLong’s test was used to compare AUCs between models. Additionally, accuracy, sensitivity, specificity, precision, recall, and F1-score were computed to comprehensively evaluate predictive performance.

The Shapley additive explanations (SHAP) algorithm was employed to interpret the best-performing model and visualize the relative contribution and direction of each predictor. A *p*-value <0.05 (two-sided) was considered statistically significant.

## Result

### General situation

A total of 301 patients with hearing loss were included, including 162 males (53.8%), 139 females (46.2%), 115 patients (38.2%) without tinnitus or mild tinnitus, and 186 patients (61.8%) with clinically significant tinnitus. The data from the training and validation sets were compared, and there was no statistically significant difference in each indicator between the two datasets (*p* > 0.05) (see Table 1). Baseline characteristics of patients with no or mild tinnitus and those with clinically significant tinnitus in the overall cohort are provided in Supplementary Table S1.

**Table 1:** Comparison of patient data between training set and validation set.

Variables	Overall (301)	Train (210)	Validation (91)	Statistic^a^	*p*-value
Age					
< 60	85 (28.2%)	59 (28.1%)	26 (28.6%)	0.01	0.933^b^
≥ 60	216 (71.8%)	151 (71.9%)	65 (71.4%)		
Sex					
Male	162 (53.8%)	112 (53.3%)	50 (54.9%)	0.07	0.797^b^
Female	139 (46.2%)	98 (46.7%)	41 (45.1%)		
Diabetes					
No	206 (68.4%)	146 (69.5%)	60 (65.9%)	0.38	0.538^b^
Yes	95 (31.6%)	64 (30.5%)	31 (34.1%)		
Hypertension					
No	185 (61.5%)	126 (60.0%)	59 (64.8%)	0.63	0.429^b^
Yes	116 (38.5%)	84 (40.0%)	32 (35.2%)		
Smoke					
No	184 (61.1%)	127 (60.5%)	57 (62.6%)	0.12	0.724^b^
Yes	117 (38.9%)	83 (39.5%)	34 (37.4%)		
Alcohol consumption					
No	114 (37.9%)	79 (37.6%)	35 (38.5%)	0.02	0.890^b^
Yes	187 (62.1%)	131 (62.4%)	56 (61.5%)		
Sleep disorder					
No	120 (39.9%)	83 (39.5%)	37 (40.7%)	0.03	0.853^b^
Yes	181 (60.1%)	127 (60.5%)	54 (59.3%)		
Anxiety					
No	147 (48.8%)	105 (50.0%)	42 (46.2%)	0.38	0.540^b^
Yes	154 (51.2%)	105 (50.0%)	49 (53.8%)		
Hearing loss severity					
Mild	125 (41.5%)	88 (41.9%)	37 (40.7%)	2.09	0.352^b^
Moderate	76 (25.2%)	57 (27.1%)	19 (20.9%)		
Severe	100 (33.2%)	65 (31.0%)	35 (38.5%)		
Duration of hearing loss					
<12 months	151 (50.2%)	107 (51.0%)	44 (48.4%)	0.17	0.679^b^
≥12 months	150 (49.8%)	103 (49.0%)	47 (51.6%)		
LPR					
No	256 (85.0%)	177 (84.3%)	79 (86.8%)	0.32	0.572^b^
Yes	45 (15.0%)	33 (15.7%)	12 (13.2%)		
Tinnitus					
No or mild	115 (38.2%)	80 (38.1%)	35 (38.5%)	0.00	0.952^b^
Moderate-to-severe	186 (61.8%)	130 (61.9%)	56 (61.5%)		
Hearing loss side					
Bilateral	161 (53.5%)	115 (54.8%)	46 (50.5%)	0.45	0.501^b^
Unilateral	140 (46.5%)	95 (45.2%)	45 (49.5%)		
Depression					
No	157 (52.2%)	107 (51.0%)	50 (54.9%)	0.41	0.524^b^
Yes	144 (47.8%)	103 (49.0%)	41 (45.1%)		
Ototoxic drug use					
No	220 (73.1%)	154 (73.3%)	66 (72.5%)	0.02	0.885^b^
Yes	81 (26.9%)	56 (26.7%)	25 (27.5%)		
Noise exposure					
No	217 (72.1%)	154 (73.3%)	63 (69.2%)	0.53	0.466^b^
Yes	84 (27.9%)	56 (26.7%)	28 (30.8%)		
Family history					
No	287 (95.3%)	200 (95.2%)	87 (95.6%)		>0.999^c^
Yes	14 (4.7%)	10 (4.8%)	4 (4.4%)		
Hyperlipidemia					
No	212 (70.4%)	149 (71.0%)	63 (69.2%)	0.09	0.764^b^
Yes	89 (29.6%)	61 (29.0%)	28 (30.8%)		
BMI					
Normal	156 (51.8%)	111 (52.9%)	45 (49.5%)		0.409^c^
Overweight	78 (25.9%)	49 (23.3%)	29 (31.9%)		
Obese	51 (16.9%)	37 (17.6%)	14 (15.4%)		
Underweight	16 (5.3%)	13 (6.2%)	3 (3.3%)		

a Pearson’s Chi-squared test; Fisher’s exact test.

b Pearson’s Chi-squared test.

c Fisher’s exact test.

LPR, laryngopharyngeal reflux; BMI, body mass index.

Univariate analysis was conducted on the training set, comparing patients with no or mild tinnitus and those with clinically significant tinnitus. As shown in Table 2, age, hypertension, sleep disorder, anxiety, hearing loss severity, depression, and noise exposure history were significantly associated with clinically significant tinnitus (*p* < 0.05).

**Table 2:** Baseline feature table of training set.

Variables	No or mild tinnitus (THI<38, *n* = 80)	Clinically significant tinnitus (THI≥38, *n* = 130)	Statistic^a^	*p*-value
Age				
< 60	32 (40.0%)	27 (20.8%)	9.07	0.003^b^
≥ 60	48 (60.0%)	103 (79.2%)		
Sex				
Male	46 (57.5%)	66 (50.8%)	0.90	0.342^b^
Female	34 (42.5%)	64 (49.2%)		
Diabetes				
No	54 (67.5%)	92 (70.8%)	0.25	0.617^b^
Yes	26 (32.5%)	38 (29.2%)		
Hypertension				
No	56 (70.0%)	70 (53.8%)	5.38	0.020^b^
Yes	24 (30.0%)	60 (46.2%)		
Smoke				
No	50 (62.5%)	77 (59.2%)	0.22	0.638^b^
Yes	30 (37.5%)	53 (40.8%)		
Alcohol consumption				
No	32 (40.0%)	47 (36.2%)	0.31	0.576^b^
Yes	48 (60.0%)	83 (63.8%)		
Sleep disorder				
No	40 (50.0%)	43 (33.1%)	5.93	0.015^b^
Yes	40 (50.0%)	87 (66.9%)		
Anxiety				
No	47 (58.8%)	58 (44.6%)	3.96	0.047^b^
Yes	33 (41.3%)	72 (55.4%)		
Hearing loss severity				
Mild	44 (55.0%)	44 (33.8%)	9.30	0.010^b^
Moderate	18 (22.5%)	39 (30.0%)		
Severe	18 (22.5%)	47 (36.2%)		
Duration of hearing loss				
<12 months	39 (48.8%)	68 (52.3%)	0.25	0.616^b^
≥12 months	41 (51.3%)	62 (47.7%)		
LPR				
No	64 (80.0%)	113 (86.9%)	1.79	0.181^b^
Yes	16 (20.0%)	17 (13.1%)		
Hearing loss side				
Bilateral	37 (46.3%)	78 (60.0%)	3.78	0.052^b^
Unilateral	43 (53.8%)	52 (40.0%)		
Depression				
No	50 (62.5%)	57 (43.8%)	6.90	0.009^b^
Yes	30 (37.5%)	73 (56.2%)		
Ototoxic drug use				
No	63 (78.8%)	91 (70.0%)	1.94	0.164^b^
Yes	17 (21.3%)	39 (30.0%)		
Noise exposure				
No	65 (81.3%)	89 (68.5%)	4.14	0.042^b^
Yes	15 (18.8%)	41 (31.5%)		
Family history				
No	75 (93.8%)	125 (96.2%)		0.510^c^
Yes	5 (6.3%)	5 (3.8%)		
Hyperlipidemia				
No	59 (73.8%)	90 (69.2%)	0.49	0.484^b^
Yes	21 (26.3%)	40 (30.8%)		
BMI				
Normal	44 (55.0%)	67 (51.5%)		0.366^c^
Overweight	22 (27.5%)	27 (20.8%)		
Obese	10 (12.5%)	27 (20.8%)		
Underweight	4 (5.0%)	9 (6.9%)		

a Pearson’s Chi-squared test; Fisher’s exact test.

b Pearson’s Chi-squared test.

c Fisher’s exact test.

LPR: laryngopharyngeal reflux, BMI: body mass index.

### Variable screening results

**Figure 1:**
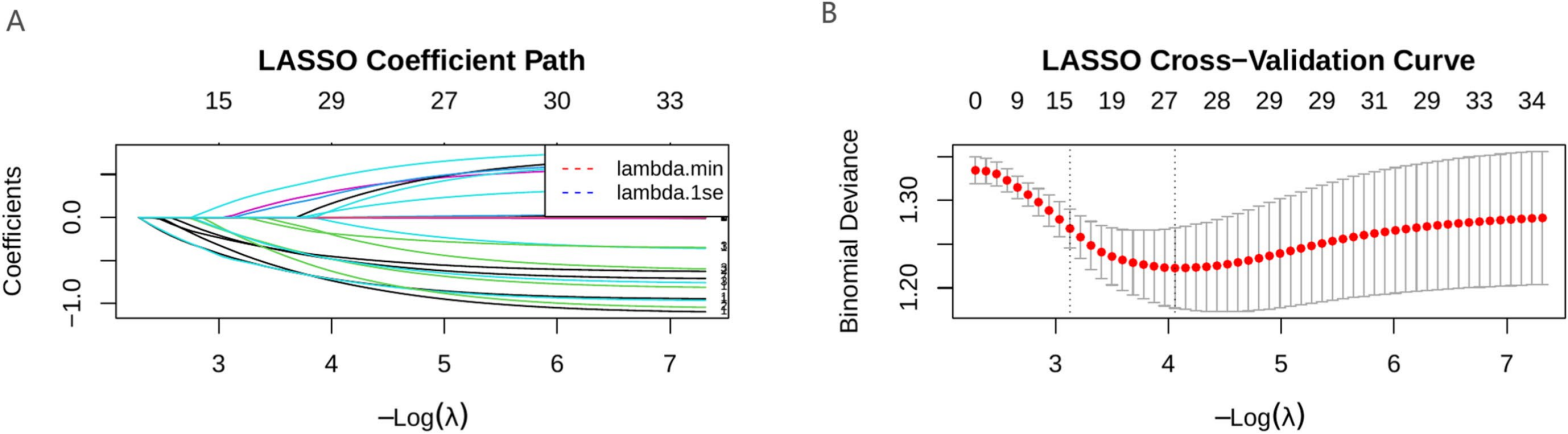
LASSO correlation graph. **(A)** LASSO coefficient path diagram; **(B)** LASSO cross-validation curve.

**Figure 2:**
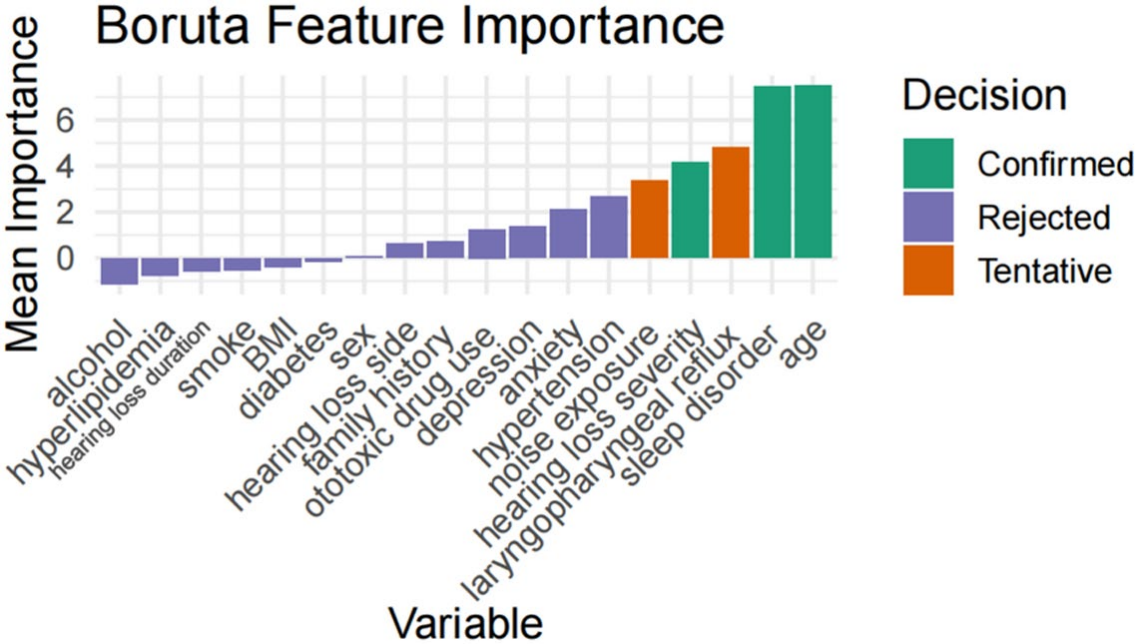
Boruta feature selection results.

LASSO regression retained nine predictive variables: age, hypertension, sleep disorder, anxiety, hearing loss severity, hearing side, depression, ototoxic drug history, and noise exposure history. The Boruta algorithm confirmed three stable predictors—age, sleep disorder, and hearing loss severity—as important features. After integrating the results from all two methods, a total of nine variables (age, hypertension, sleep disorder, anxiety, hearing loss severity, depression, noise exposure history, hearing side, and ototoxic drug history) were selected for subsequent model construction (Figures 1, 2).

### Model performance comparison

**Figure 3:**
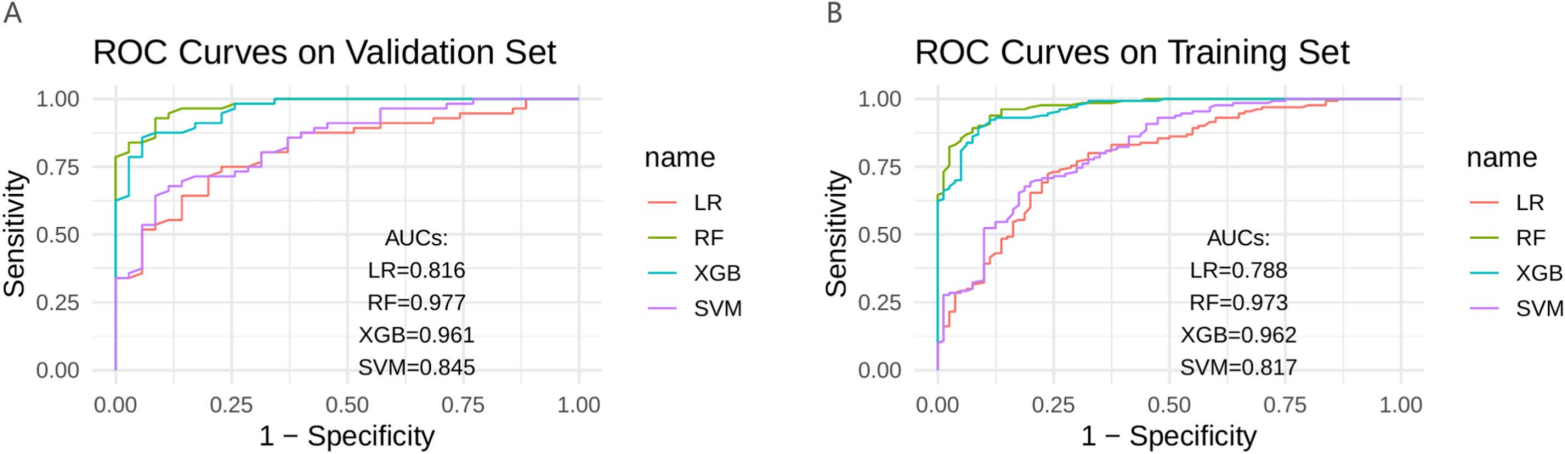
Four models in the training set and validate the ROC curve in the set. **(A)** Validation set; **(B)** Train set; ROC, receiver operating characteristic curve; AUC, area under the curve; LR, logistic regression; RF, random forest; XGB, extreme gradient boosting; SVM, support vector machine.

In the training set, the RF model exhibited the best predictive ability (AUC = 0.973), followed by XGB (AUC = 0.962), LR (AUC = 0.788), and SVM (AUC = 0.817). In the validation set, the RF model again achieved the highest discrimination (AUC = 0.977), followed by XGB (AUC = 0.961), LR (AUC = 0.816), and SVM (AUC = 0.845) (Figure 3). The DeLong test was performed to statistically compare the AUCs among the four models. In both the training and validation sets, the RF model achieved the highest AUC values and demonstrated significantly superior discrimination compared with LR (*p* < 0.001) and SVM (*p* < 0.001). However, the differences between RF and XGB were not statistically significant (*p* = 0.055 in the training set; *p* = 0.075 in the validation set) (Table 3).

**Table 3:** AUC comparison and DeLong test results of four models in the training set and validation set.

Dataset	Model	AUC (95% CI)	Comparison (RF vs.)	*Z* value	*p*-value
Training	RF	0.973 (0.961–0.985)	–	–	–
	LR	0.788 (0.742–0.834)	RF vs. LR	6.39	1.66 × 10^−10^
	XGB	0.962 (0.948–0.975)	RF vs. XGB	1.92	0.055
	SVM	0.817 (0.773–0.861)	RF vs. SVM	5.95	2.76 × 10^−9^
Validation	RF	0.977 (0.963–0.991)	–	–	–
	LR	0.816 (0.764–0.868)	RF vs. LR	4.14	3.49 × 10^−5^
	XGB	0.961 (0.945–0.978)	RF vs. XGB	1.78	0.075
	SVM	0.845 (0.791–0.898)	RF vs. SVM	3.84	1.22 × 10^−4^

AUC, area under the curve; LR, logistic regression; RF, random forest; XGB, extreme gradient boosting; SVM, support vector machine.

Evaluate the performance of the model using four indicators: accuracy, precision, recall, and F1 score (25). The predictive performance of the four ML models in both the training and validation datasets is summarized in Table 4.

**Table 4:** Comparison of performance evaluation of four models in training and validation sets.

Dataset	Model	Accuracy	Sensitivity	Specificity	Precision	Recall	F1-score
Training	LR	0.752	0.831	0.625	0.783	0.831	0.806
	RF	0.914	0.962	0.838	0.906	0.962	0.933
	XGB	0.905	0.931	0.862	0.917	0.931	0.924
	SVM	0.767	0.908	0.538	0.761	0.908	0.828
Validation	LR	0.769	0.857	0.629	0.787	0.857	0.821
	RF	0.923	0.929	0.914	0.945	0.929	0.937
	XGB	0.890	0.875	0.914	0.942	0.875	0.907
	SVM	0.758	0.875	0.571	0.766	0.875	0.817

In the training set, RF model achieved the highest overall performance, with an accuracy of 0.914, sensitivity of 0.962, specificity of 0.838, precision of 0.906, recall of 0.962, and F1-score of 0.933. XGB model demonstrated comparable performance (accuracy = 0.905, F1-score = 0.924). LR and SVM models showed relatively lower accuracies of 0.752 and 0.767, respectively.

In the validation set, the RF model again performed best, with an accuracy of 0.923, sensitivity of 0.929, specificity of 0.914, precision of 0.945, recall of 0.929, and F1-score of 0.937. The XGB model also exhibited high predictive ability (accuracy = 0.890, F1-score = 0.907), whereas LR and SVM had moderate predictive performance (accuracies of 0.769 and 0.758, respectively).

Overall, the RF model achieved the most balanced and robust performance across both datasets, showing superior discrimination and generalization ability compared with other algorithms.

**Figure 4:**
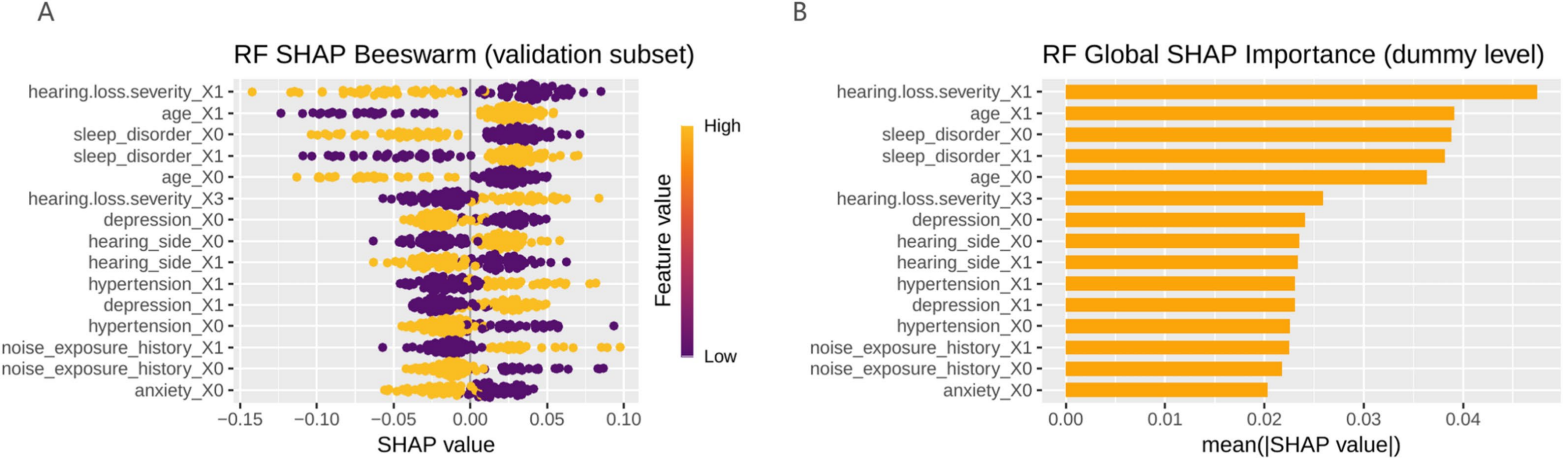
The global SHAP summary plot. **(A)** Model feature bee colony diagram (the color gradient represents feature magnitude, with yellow indicating higher feature values and purple indicating lower feature values); **(B)** Bar chart of model features; RF, random forest; SHAP, Shapley additive explanations.

Perform interpretability analysis on the model based on SHAP. For each patient, the contribution of each predictor variable to the final outcome is represented by the SHAP value, with a higher SHAP value indicating a higher likelihood of the patient experiencing clinically significant tinnitus. The global SHAP summary plot (Figure 4) demonstrates that hearing loss severity, age, and sleep disorder were the most influential predictors, followed by depression, hearing side, hypertension, and noise exposure history.

These findings suggest that more severe hearing loss, older age, and poor sleep quality were strongly associated with an increased likelihood of developing clinically significant tinnitus.

## Discussion

In this study, we developed and validated several ML models—including RF, XGBoost, SVM, and LR—to predict clinically significant tinnitus in patients with hearing loss. Our models achieved robust predictive performance, accurately distinguishing patients at risk of significant tinnitus symptoms. Greater hearing loss severity emerged as a top predictor, consistent with prior large-scale analyses suggesting that hearing impairment is an important risk factor for the development of more severe tinnitus, with population-based models indicating that hearing-related variables contribute substantially to the prediction of tinnitus severity (11, 26). In addition, a range of comorbid conditions—particularly sleep disturbances (e.g., insomnia or poor sleep quality) and psychological symptoms such as depression and anxiety—were strongly associated with higher tinnitus severity (27, 28). These non-auditory factors significantly improved the model’s ability to flag high-risk patients, underlining that tinnitus severity is a multi-factorial outcome. For example, patients reporting frequent fatigue, poor sleep, and “gloomy” mood (indicative of depression) were far more likely to have severe tinnitus. This is consistent with recent research on large-scale populations (14, 29). Among the ML algorithms, ensemble tree-based models (like RF and XGBoost) performed particularly well, suggesting that complex, non-linear interactions among variables were successfully captured. When evaluating model performance using F1 scores from the validation set, the RF model exhibits the most balanced performance through joint optimization of accuracy and recall. This result is in line with other studies where advanced classifiers yielded high accuracy in tinnitus classification; for instance, combining audiometric data with clinical features via an SVM has achieved area-under-curve values around 0.9 in classifying tinnitus distress levels (30). Overall, our findings highlight that an integrative ML approach can effectively identify hearing-impaired patients at risk for clinically significant tinnitus, with hearing loss and comorbid factors jointly driving risk.

Depression, anxiety, and sleep disorders exert a strong influence on tinnitus severity, indicating shared pathophysiological mechanisms between emotional regulation, sleep, and tinnitus perception (31). Tinnitus is now recognized as a disorder involving broader neural networks beyond the auditory system, particularly those related to stress and emotion. Psychological distress is common in chronic tinnitus—up to 60% of patients experience depression, 30–45% report anxiety, and many suffer from insomnia or poor sleep quality (32). These comorbidities not only result from intrusive tinnitus but also amplify its perceived loudness and distress. A vicious cycle may occur: tinnitus triggers stress and negative emotions, which further enhance tinnitus perception through increased limbic and attentional activity (33). Abnormal hypothalamic–pituitary–adrenal (HPA) axis responses and autonomic arousal have also been observed, suggesting dysregulated stress physiology. Poor sleep and fatigue exacerbate this loop, as severe tinnitus patients often show shorter sleep duration and more insomnia (34, 35). Overall, tinnitus severity depends not only on auditory damage but also on emotional and cognitive factors, resembling chronic pain mechanisms. Interventions addressing these non-auditory components—such as improving sleep, treating depression, or correcting hearing loss—can alleviate both tinnitus distress and associated psychological symptoms.

Our study adds to a growing body of research applying ML to tinnitus prediction and management. Historically, clinicians have relied on audiological exams and self-report questionnaires to assess tinnitus, but these methods alone often miss the multidimensional nature of tinnitus severity (36). The use of ML models represents a novel and valuable approach—one that can integrate diverse data (hearing thresholds, psychiatric history, sleep quality, etc.) to improve risk stratification. Recent studies underscore this potential. For instance, Jafari et al. (37) demonstrated that neural network and RF models could successfully differentiate tinnitus sufferers and types of hearing loss, pointing to the feasibility of incorporating ML into routine audiology practice. Recent studies have also explored alternative ML paradigms in hearing research. For example, Yang et al. (38) proposed a soft classification framework based on quadratic discriminant analysis to probabilistically characterize heterogeneous audiometric phenotypes of age-related hearing loss. This approach provides a more nuanced description of hearing phenotypes by allowing individuals to partially belong to multiple classes rather than forcing a single hard classification. In our context, the ability to predict which hearing-impaired patients are likely to develop clinically significant tinnitus has clear clinical importance. Such prognostic insight allows for proactive intervention: patients flagged as high-risk could receive earlier counseling, psychological support, or sound therapies to possibly prevent escalation of tinnitus distress. The present findings particularly highlight the benefit of a multidimensional assessment. By concurrently evaluating audiometric injury (the “input” driving tinnitus) and factors like mood disturbances or poor sleep (which shape the “reaction” to tinnitus), clinicians can obtain a more complete risk profile. This aligns with emerging holistic models of tinnitus care, which emphasize that effective management should address not only the peripheral ear damage but also the patient’s mental health and sleep hygiene (39). The application of ML in this domain is novel in that it provides an objective, data-driven method to combine these elements. It moves tinnitus screening beyond one-size-fits-all metrics toward personalized medicine—for example, an ML-based tool could be used in hearing clinics to automatically flag patients who, based on their hearing loss and questionnaire scores (for insomnia, depression, etc.), are likely to experience severe tinnitus. This approach complements current treatment guidelines that already advocate interdisciplinary management (including cognitive-behavioral therapy for tinnitus-related distress) (40). Together, these advancements illustrate a new paradigm: leveraging ML to integrate audiological and psychosocial dimensions for improved tinnitus risk prediction, thereby enhancing clinical decision-making and patient outcomes.

## Limitations

This study has several limitations. First, its retrospective, single-center design limits generalizability. The model was developed in a specific population and lacks external validation, so its performance in other cohorts remains uncertain. Future research should include multi-center data and external validation to improve robustness. Second, the cross-sectional nature of our analysis prevents causal inference and long-term observation. Longitudinal studies are needed to clarify temporal relationships—such as whether depression or poor sleep precede tinnitus worsening—and to identify long-term risk factors. Third, most variables (e.g., sleep disturbance, anxiety, depression) were self-reported, which may introduce bias. Future studies should incorporate objective assessments such as actigraphy, polysomnography, or structured psychiatric evaluations, along with biological markers (e.g., stress hormones or neuroimaging). Moreover, the relatively small sample size may have limited model complexity. Larger datasets could enable the use of advanced or deep learning models to enhance predictive performance. Finally, while our model identified associations, its clinical utility remains to be tested. Prospective implementation studies are required to determine whether applying such models improves early identification and management of patients at high risk for severe tinnitus (36, 37).

## Conclusion

The RF model demonstrated superior predictive accuracy for identifying patients with hearing loss at risk of clinically significant tinnitus. Key predictors included hearing loss severity, age, and sleep disorder, indicating that integrating auditory and psychological factors enhances risk assessment and supports early, individualized intervention strategies.

## Data Availability

The raw data supporting the conclusions of this article will be made available by the authors, without undue reservation.
