# Supplementary material for: Development and comparison of machine learning models for predicting moderate-to-severe tinnitus in patients with hearing loss
Source: Front Neurol. 2026 Jan 12;16:1741302. doi: 10.3389/fneur.2025.1741302 (PMC12832511; doi:10.3389/fneur.2025.1741302)
Supplement: Supplementary file 1 [file Table_1.DOCX]

**Table S1 Baseline characteristics of patients**

| **Variables** | **Total (n = 301)** | **Clinically significant Tinnitus (THI ≥ 38, n = 186)** | **No or Mild Tinnitus (THI < 38, n = 115)** | **p-value** |
| --- | --- | --- | --- | --- |
| age |  |  |  | 0.002* |
| < 60 | 85 (28.2%) | 40 (21.5%) | 45 (39.1%) |  |
| ≥ 60 | 216 (71.8%) | 146 (78.5%) | 70 (60.9%) |  |
| sex |  |  |  | 0.702 |
| Male | 162 (53.8%) | 98 (52.7%) | 64 (55.7%) |  |
| Female | 139 (46.2%) | 88 (47.3%) | 51 (44.3%) |  |
| diabetes |  |  |  | 0.283 |
| No | 206 (68.4%) | 132 (71.0%) | 74 (64.3%) |  |
| Yes | 95 (31.6%) | 54 (29.0%) | 41 (35.7%) |  |
| hypertension |  |  |  | 0.004* |
| No | 185 (61.5%) | 102 (54.8%) | 83 (72.2%) |  |
| Yes | 116 (38.5%) | 84 (45.2%) | 32 (27.8%) |  |
| smoke |  |  |  | 0.206 |
| No | 184 (61.1%) | 108 (58.1%) | 76 (66.1%) |  |
| Yes | 117 (38.9%) | 78 (41.9%) | 39 (33.9%) |  |
| alcohol consumption |  |  |  | 0.471 |
| No | 114 (37.9%) | 67 (36.0%) | 47 (40.9%) |  |
| Yes | 187 (62.1%) | 119 (64.0%) | 68 (59.1%) |  |
| sleep disorder |  |  |  | 0.005* |
| No | 120 (39.9%) | 62 (33.3%) | 58 (50.4%) |  |
| Yes | 181 (60.1%) | 124 (66.7%) | 57 (49.6%) |  |
| anxiety |  |  |  | 0.007* |
| No | 147 (48.8%) | 79 (42.5%) | 68 (59.1%) |  |
| Yes | 154 (51.2%) | 107 (57.5%) | 47 (40.9%) |  |
| hearing loss severity |  |  |  | <0.001* |
| Mild | 125 (41.5%) | 59 (31.7%) | 66 (57.4%) |  |
| Moderate | 76 (25.2%) | 53 (28.5%) | 23 (20.0%) |  |
| Severe | 100 (33.2%) | 74 (39.8%) | 26 (22.6%) |  |
| duration of hearing loss |  |  |  | 0.778 |
| <12 months | 151 (50.2%) | 95 (51.1%) | 56 (48.7%) |  |
| ≥12 months | 150 (49.8%) | 91 (48.9%) | 59 (51.3%) |  |
| LPR |  |  |  | 0.152 |
| No | 256 (85.0%) | 163 (87.6%) | 93 (80.9%) |  |
| Yes | 45 (15.0%) | 23 (12.4%) | 22 (19.1%) |  |
| hearing loss side |  |  |  | 0.017* |
| Bilateral | 161 (53.5%) | 110 (59.1%) | 51 (44.3%) |  |
| Unilateral | 140 (46.5%) | 76 (40.9%) | 64 (55.7%) |  |
| depression |  |  |  | 0.001* |
| No | 157 (52.2%) | 83 (44.6%) | 74 (64.3%) |  |
| Yes | 144 (47.8%) | 103 (55.4%) | 41 (35.7%) |  |
| ototoxic drug use |  |  |  | 0.234 |
| No | 220 (73.1%) | 131 (70.4%) | 89 (77.4%) |  |
| Yes | 81 (26.9%) | 55 (29.6%) | 26 (22.6%) |  |
| noise exposure |  |  |  | 0.081 |
| No | 217 (72.1%) | 127 (68.3%) | 90 (78.3%) |  |
| Yes | 84 (27.9%) | 59 (31.7%) | 25 (21.7%) |  |
| family history |  |  |  | 0.226 |
| No | 287 (95.3%) | 180 (96.8%) | 107 (93.0%) |  |
| Yes | 14 (4.7%) | 6 (3.2%) | 8 (7.0%) |  |
| hyperlipidemia |  |  |  | 0.896 |
| No | 212 (70.4%) | 130 (69.9%) | 82 (71.3%) |  |
| Yes | 89 (29.6%) | 56 (30.1%) | 33 (28.7%) |  |
| BMI |  |  |  | 0.104 |
| Normal | 156 (51.8%) | 92 (49.5%) | 64 (55.7%) |  |
| Overweight | 78 (25.9%) | 44 (23.7%) | 34 (29.6%) |  |
| Obese | 51 (16.9%) | 38 (20.4%) | 13 (11.3%) |  |
| Underweight | 16 (5.3%) | 12 (6.5%) | 4 (3.5%) |  |

Note: BMI: body mass index; LPR: laryngopharyngeal reflux; THI: Tinnitus Handicap Inventory; Variables with p-value < 0.05 were considered statistically significant and are marked with *.
